# Artificial intelligence large language model ChatGPT: is it a trustworthy and reliable source of information for sarcoma patients?

**DOI:** 10.3389/fpubh.2024.1303319

**Published:** 2024-03-22

**Authors:** Marisa Valentini, Joanna Szkandera, Maria Anna Smolle, Susanne Scheipl, Andreas Leithner, Dimosthenis Andreou

**Affiliations:** ^1^Department of Orthopaedics and Trauma, Medical University of Graz, Graz, Austria; ^2^Division of Oncology, Department of Internal Medicine, Medical University of Graz, Graz, Austria

**Keywords:** artificial intelligence, ChatGPT, sarcoma, patient information, information quality

## Abstract

**Introduction:**

Since its introduction in November 2022, the artificial intelligence large language model ChatGPT has taken the world by storm. Among other applications it can be used by patients as a source of information on diseases and their treatments. However, little is known about the quality of the sarcoma-related information ChatGPT provides. We therefore aimed at analyzing how sarcoma experts evaluate the quality of ChatGPT’s responses on sarcoma-related inquiries and assess the bot’s answers in specific evaluation metrics.

**Methods:**

The ChatGPT responses to a sample of 25 sarcoma-related questions (5 definitions, 9 general questions, and 11 treatment-related inquiries) were evaluated by 3 independent sarcoma experts. Each response was compared with authoritative resources and international guidelines and graded on 5 different metrics using a 5-point Likert scale: completeness, misleadingness, accuracy, being up-to-date, and appropriateness. This resulted in maximum 25 and minimum 5 points per answer, with higher scores indicating a higher response quality. Scores ≥21 points were rated as very good, between 16 and 20 as good, while scores ≤15 points were classified as poor (11–15) and very poor (≤10).

**Results:**

The median score that ChatGPT’s answers achieved was 18.3 points (IQR, i.e., Inter-Quartile Range, 12.3–20.3 points). Six answers were classified as very good, 9 as good, while 5 answers each were rated as poor and very poor. The best scores were documented in the evaluation of how appropriate the response was for patients (median, 3.7 points; IQR, 2.5–4.2 points), which were significantly higher compared to the accuracy scores (median, 3.3 points; IQR, 2.0–4.2 points; *p* = 0.035). ChatGPT fared considerably worse with treatment-related questions, with only 45% of its responses classified as good or very good, compared to general questions (78% of responses good/very good) and definitions (60% of responses good/very good).

**Discussion:**

The answers ChatGPT provided on a rare disease, such as sarcoma, were found to be of very inconsistent quality, with some answers being classified as very good and others as very poor. Sarcoma physicians should be aware of the risks of misinformation that ChatGPT poses and advise their patients accordingly.

## Introduction

Sarcomas are a heterogeneous group of rare malignant tumors, accounting for merely 1% of all cancer diagnoses (1). Their overall incidence is estimated at approximately 7.1–7.4 per 100,000 patients per year (1, 2). Due to their rarity and complexity, international guidelines recommend a multidisciplinary diagnostic and therapeutic approach at specialized sarcoma centers (3–6). Finding accurate and reliable information can be challenging for patients, caregivers, and healthcare professionals who are not specialized in sarcoma treatment (7).

Since its introduction in November 2022, the Artificial Intelligence (AI) Chat Generative Pre-trained Transformer (ChatGPT) has taken the world by storm (8–12). ChatGPT is a 175-billion-parameter natural language processing model (GPT 3.5), able to generate conversation-style responses to user input (10). As a large language model trained on a massive dataset of text and data available online, it is able to generate responses to a wide range of questions (13). Due to its very nature, the artificial intelligence chatbot can address an almost limitless range of inquiries, but it is not capable of verifying the accuracy of its responses and may not provide the most up-to-date or comprehensive information. Among other applications, it has been used and will likely be increasingly used by patients as a source of information on diseases and their treatments, but its potential to generate inaccurate or false information is a major cause for concern (14–18). Previous studies have shown that the bot may be a useful source of information for common rheumatic diseases (19) and provide more empathetic answers to general public questions compared to physicians (14), but also demonstrated that the quality of the bot’s responses is worse when confronted with more complex medical questions (20, 21).

Very little is known about the quality of the sarcoma-related information ChatGPT provides. The authors chose to focus on this rare group of tumors, as their complexity and the lack of safe online information on this topic are well known and a cause for concern (22–24). Therefore, we aimed at evaluating how complete, misleading, accurate, up-to-date, and appropriate the Open AI chatbot’s answers to sarcoma-related inquiries are, assessing the quality of the information it imparts. Specifically, we analyzed how sarcoma experts evaluate the quality of ChatGPT’s responses on sarcoma-related inquiries, how the bot’s responses perform in specific metrics of the evaluation, and if ChatGPT fares better with a specific type of questions.

## Materials and methods

A sample of 25 representative sarcoma-related questions were posed to ChatGPT (*ChatGPT 3.5 free version*) (Table 1). These included 5 definitions (e.g., what is a tenosynovial giant cell tumor?), 9 general questions (e.g., which imaging modalities are best in follow-up after treatment of soft tissue sarcoma, or what are common side effects of chemotherapy for Ewing sarcoma?), and 11 treatment-related inquiries (e.g., what is the optimal treatment of a desmoid tumor?). Three sarcoma experts (2 orthopedic oncologists and 1 medical oncologist) evaluated the artificial intelligence chatbot’s responses, comparing them to international guidelines and authoritative resources. The evaluation was performed independently by and without contact between these experts. Each response was graded with regards to 5 different aspects/evaluation metrics, using a 5-point Likert scale: completeness, misleadingness, accuracy (i.e., whether the response contained relevant factual errors), being up-to-date, and appropriateness (i.e., whether it’d be a good source of information for patients) (Table 2). This resulted in a maximum of 25 and a minimum of 5 points per answer, with higher scores indicating higher quality of the ChatGPT response. Scores ≥21 were defined as very good, between 16 and 20 points as good, while responses that scored less than 15 points were classified as poor (11–15) and very poor (≤10).

**Table 1:** The 25 sarcoma-related questions which were posed to ChatGPT.

**N°**	**Questions**
1	What is the optimal treatment of a desmoid tumor?
2	What is the optimal treatment of Ewing sarcoma?
3	What are the most helpful chemotherapeutic agents in the treatment of Ewing sarcoma?
4	Is follow-up necessary after treatment of soft tissue sarcoma?
5	Which imaging modalities are best in follow-up after treatment of soft tissue sarcoma?
6	What is the preferred surgical treatment for clear cell chondrosarcoma?
7	What is the optimal treatment of a retroperitoneal liposarcoma?
8	Is preoperative radiotherapy better than postoperative radiotherapy in patients with myxoid liposarcoma?
9	What late effects are possible after successful multidisciplinary treatment of osteosarcoma?
10	How can I enroll in a clinical trial for Ewing sarcoma?
11	Which clinical trials are available for Ewing sarcoma in Germany?
12	What is a biopsy for Ewing sarcoma?
13	What is the difference between enchondromas and atypical cartillaginous tumors?
14	What are common side effects of chemotherapy for Ewing sarcoma?
15	What is the difference between a lipoma and an atypical lipomatous tumor?
16	What is a tenosynovial giant cell tumor?
17	I have a Ewing sarcoma of the upper thigh bone. What is my prognosis?
18	Is an allograft-prosthetic-composite better than a megaprosthesis for an osteosarcoma of the proximal tibia?
19	What is a rotationplasty?
20	Is rotationplasty better or worse than above-knee amputation for osteosarcoma?
21	When is postoperative radiotherapy recommended for Ewing sarcoma?
22	When is postoperative radiotherapy recommended for osteosarcoma?
23	What are the advantages and disadvantages of preoperative denosumab treatment for giant cell tumor of bone?
24	What functional outcome can be expected after proximal humerus replacement with megaprosthesis for osteosarcoma?
25	What is the best treatment for gastrointestinal stromal tumors?

**Table 2:** The aspects of each ChatGPT response that were evaluated using a 5-point Likert scale.

Evaluated aspects		Score				
1	Is the provided information complete?	**5** *strongly agree*	**4** *agree*	**3** *neutral*	**2** *disagree*	**1** *strongly disagree*
2	Is the provided answer misleading?	**1** *strongly agree*	**2** *agree*	**3** *neutral*	**4** *disagree*	**5** *strongly disagree*
3	Are there relevant factual errors in the provided information?	**1** *strongly agree*	**2** *agree*	**3** *neutral*	**4** *disagree*	**5** *strongly disagree*
4	Is the provided information up to date?	**5** *strongly agree*	**4** *agree*	**3** *neutral*	**2** *disagree*	**1** *strongly disagree*
5	Is the provided answer a good source of information for patients?	**5** *strongly agree*	**4** *agree*	**3** *neutral*	**2** *disagree*	**1** *strongly disagree*

An approval from our local ethic committee was not required, as the study did not involve human subjects.

Statistical analyses were performed with Stata Version 16.1 for Mac (*StataCorp, College Station, TX, US*). Continuous variables were checked for normality with the Shapiro–Wilk test. Median values with the respective Inter-Quartile Ranges (IQR) were reported for non-normally distributed variables. The values of different aspects of a ChatGPT response were compared with the Wilcoxon signed-rank test. The overall scores of the responses in the three pre-defined categories (definitions, general questions, and treatment-related inquiries) were compared with Kruskal-Wallis and post-hoc Dunn tests. A *p*-value of <0.05 was considered significant.

## Results

**Figure 1:**
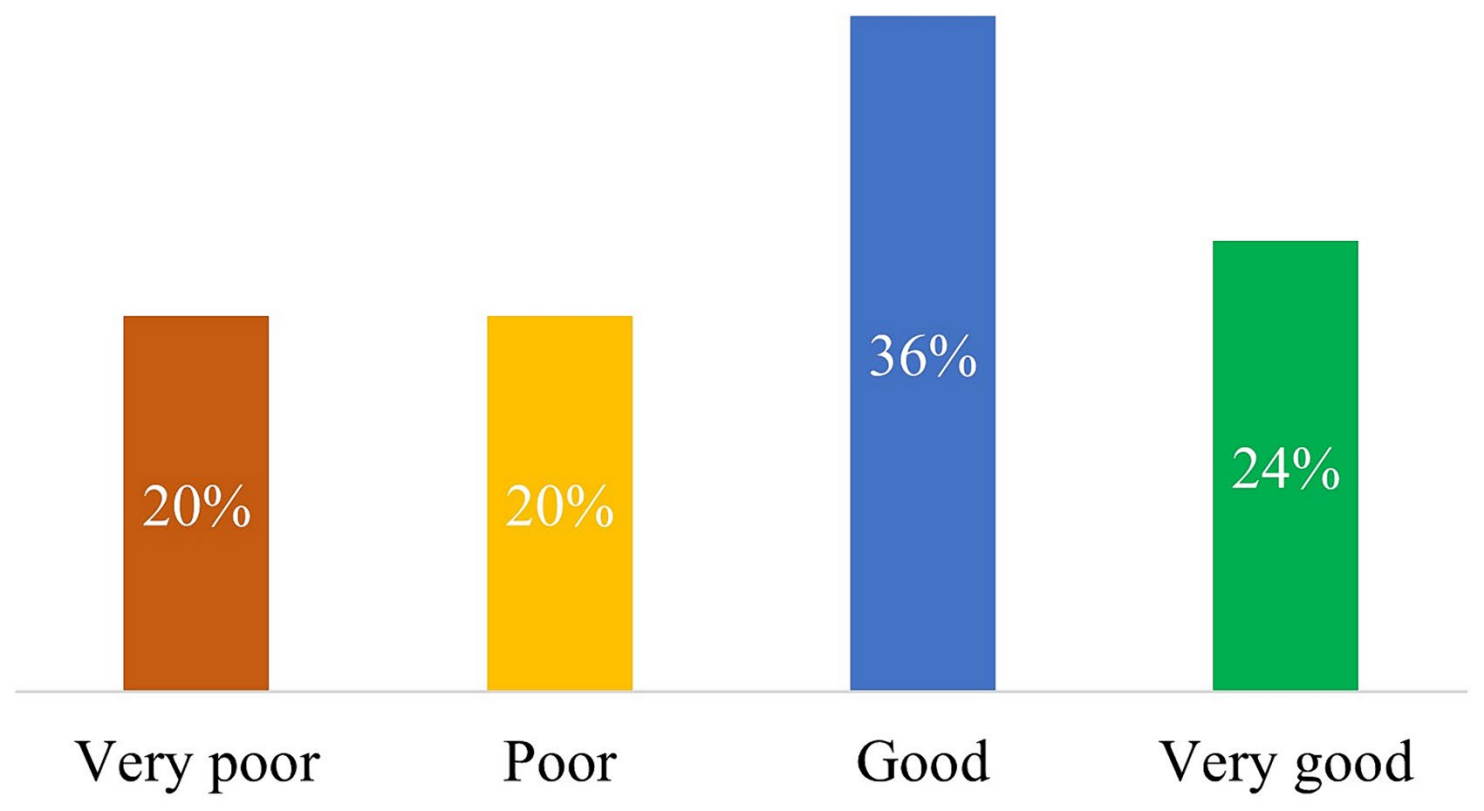
This figure depicts the evaluation of the quality of ChatGPT responses by sarcoma experts. The percentage value in each bar is based on the total number of questions (25).

The ChatGPT responses achieved a median score of 18.3 points (IQR, 12.3–20.3 points). The individual scores of each of the 5 evaluated aspects amounted to a median of 3.5 points (IQR, 2.4–4.2 points). Six of the 25 responses (24%) were classified as very good, 9/25 (36%) as good, while 5/25 answers each (20%) were defined as poor and very poor, respectively (Figure 1).

**Figure 2:**
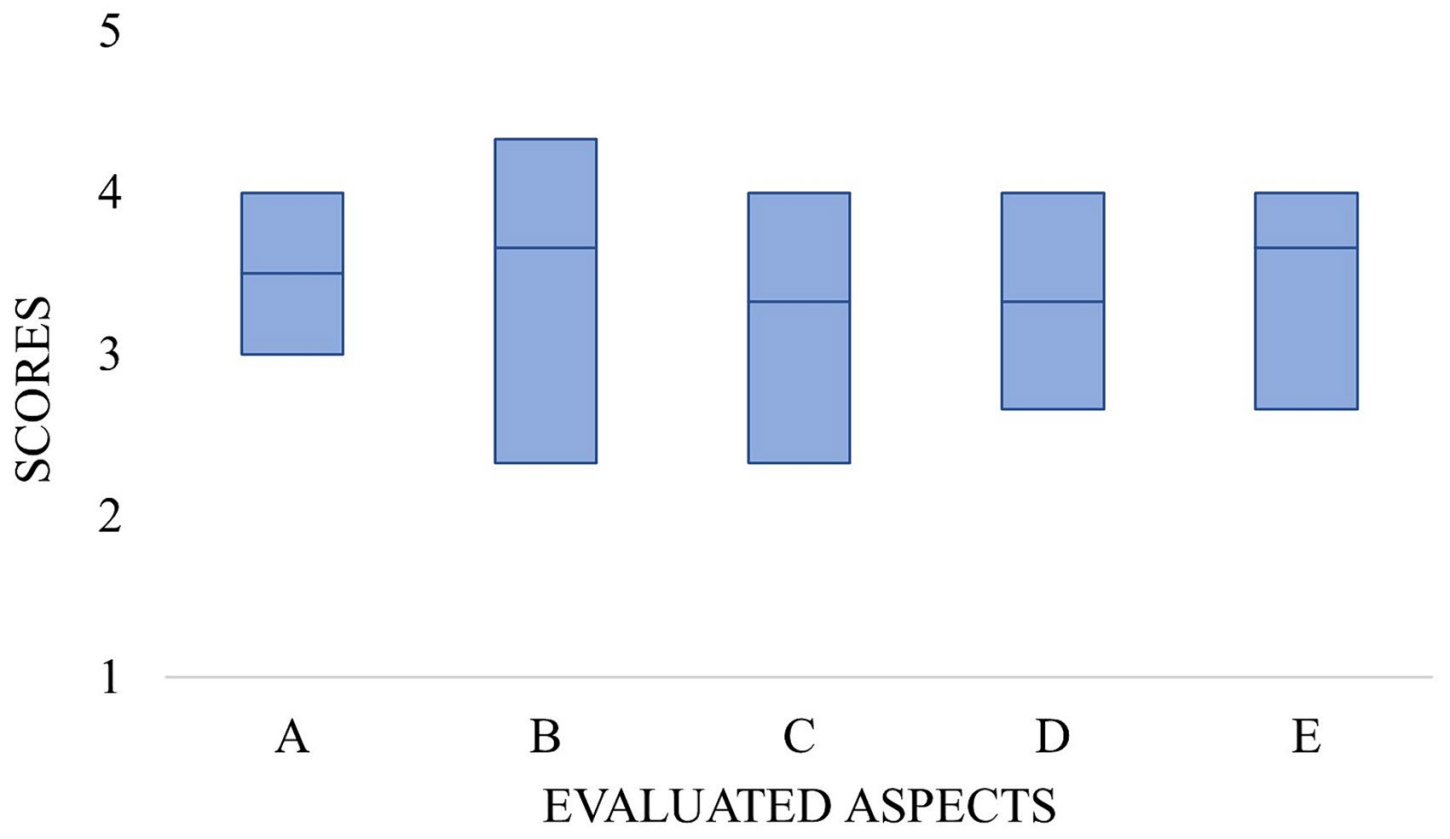
The graph shows the scores that the ChatGPT responses achieved in each specific metric of the evaluation. On the X axis the individual metrics are presented as A (completeness), B (misleadingness), C (accuracy), D (being up-to-date), and E (appropriateness). The Y axis shows the score per aspect on a 5-point Likert scale with the respective medians and IQRs.

Concerning the 5 evaluated aspects, the best scores (Figure 2) were recorded in the evaluation metric of how appropriate the response was for patients (median, 3.7 points; IQR, 2.5–4.2 points), which were significantly higher compared to the accuracy scores (median, 3.3 points; IQR, 2.0–4.2 points; *p* = 0.035). On the other hand, with the numbers we had the differences between the accuracy and completeness scores (median, 3.5 points; IQR, 2.8–4.0; *p* = 0.066) did not reach statistical significance. The remaining comparisons between the evaluation metrics showed no statistically significant differences.

**Figure 3:**
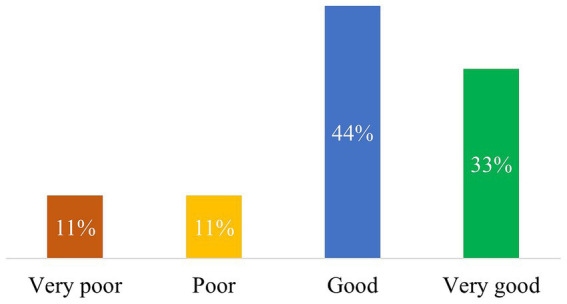
This figure shows the evaluation of the quality of ChatGPT responses to general questions. The percentage value in each bar is based on the total number of questions in this category (9).

**Figure 4:**
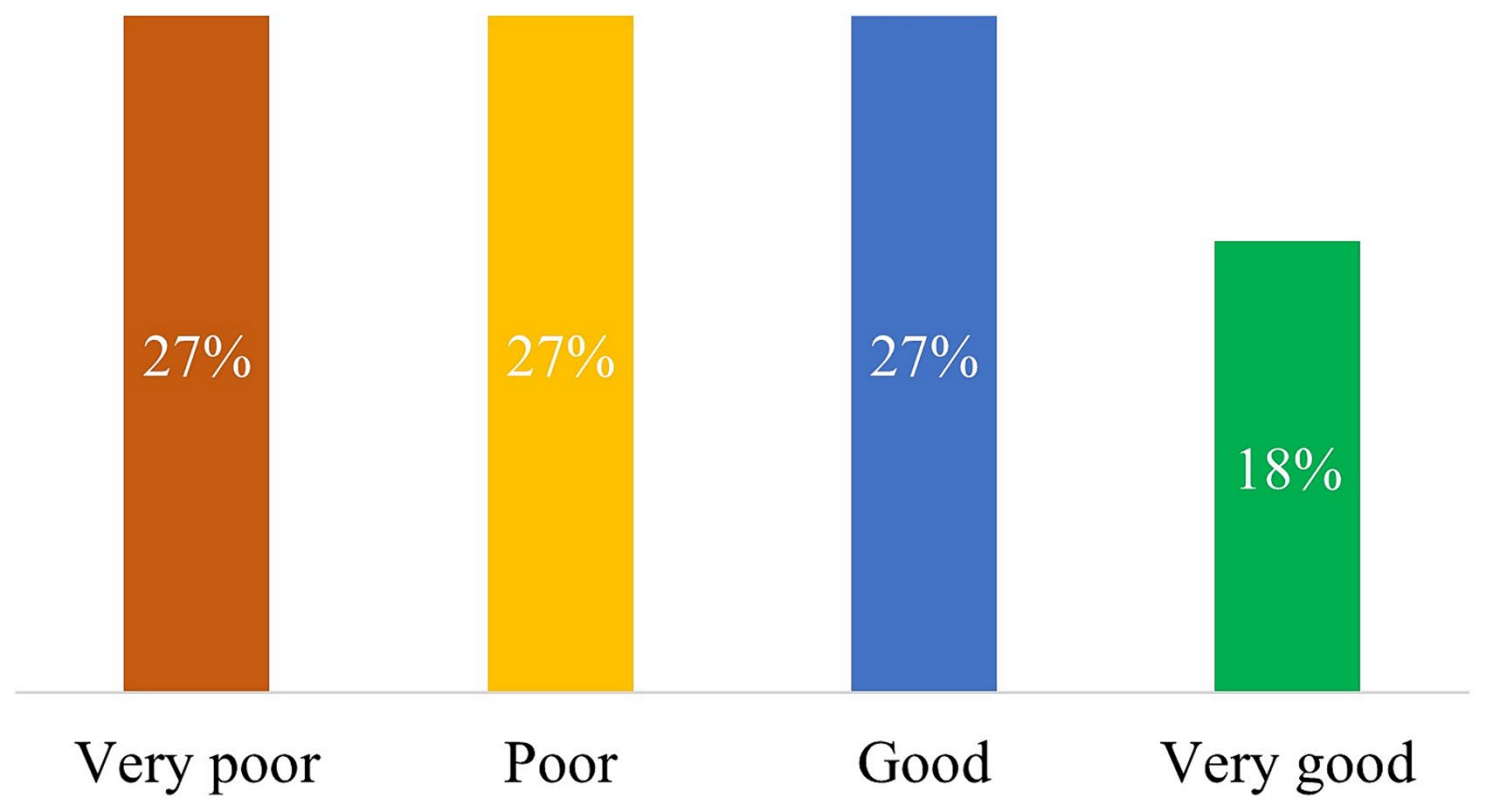
This figure shows the evaluation of the quality of ChatGPT responses to treatment related questions. The percentage value in each bar is based on the total number of questions in this category (11).

As for the 3 categories of questions, ChatGPT fared best with general inquiries, achieving good and very good overall scores in 3/9 (33%) and 4/9 (44%) questions, respectively. Only 1/9 (11%) response each was rated as poor and very poor, respectively (Figure 3). On the other hand, the bot fared considerably worse on treatment-related questions, achieving good and very good overall scores in 3/11 (27%) and 2/11 (18%), respectively. 3/11 (27%) responses each were classified as poor and very poor, respectively (Figure 4). However, with the numbers available for this analysis, these differences did not reach statistical significance (*p* = 0.063). Finally, the bot’s responses on definitions ranged better than treatment-related replies; 60% of the ChatGPT responses were classified as good (2/5, 40%) or very good (1/5, 20%), while 1/5 (20%) response each was classified as poor and very poor, respectively. No statistical significance was detected in this case as well.

## Discussion

Based on the extraordinary popularity the artificial intelligence bot ChatGPT achieved in only a few months, it is expected to quickly become an everyday health information source for patients (8, 14–17). However, little is known about the quality of information it can provide regarding rare diseases, such as sarcoma. Our study demonstrated that the responses provided by ChatGPT to sarcoma-related questions were very inconsistent in quality, ranging from very good to very poor ones. The responses scored better in the metric of appropriateness for patients and worse in their accuracy, while the bot generally fared better with general questions and worse with specific treatment-related inquiries.

We acknowledge that our study has several limitations. First of all, given the variety in presentation, prognosis, and treatment of bone and soft tissue sarcomas, our sample of 25 questions cannot be expected to cover all aspects of these rare diseases. However, we deliberately opted for a relatively small sample to avoid a bloated analysis, while the individual questions were carefully chosen based on our clinical experience to be representative of the wide range of questions patients, relatives, or caregivers might ask the Open AI chatbot. Another possible limitation of our study is that ChatGPT 3.5 was used. This free version of the AI model was trained on a massive dataset of information before its release in November 2022 and does not undergo regular updates. A newer GPT 4 model was released in March 2023. Its enhanced capabilities include being a multimodal model, taking also images as input, and the ability to interact with external interfaces. On the other hand, it needs to be considered that ChatGPT has 180.5 million active users, but only an estimated 1% subscribe to “ChatGPT Plus” (giving access to the GPT 4 model for 20$/month).^1^ This aspect of the accessibility and actual use of the GPT 4 (paid version) is of great importance. Most patients with sarcoma will most likely access the free version (ChatGPT 3.5) to seek information. Therefore, the authors believe that this study’s results are relevant as they are based on the ChatGPT version that most patients and their relatives will actually use. As such, our results reflect the information most patients will receive through the free model. Furthermore, it is not guaranteed that the GPT 4 model provides more accurate information in a rare disease, such as sarcoma, taking into consideration the long-known problem of inaccurate, outdated, and misleading sarcoma information even in reputable online sources (22–24).

The overall quality of ChatGPT responses on sarcoma-related inquiries in our study varied from very good to very poor. This variability harbors a great risk for patients in case they use ChatGPT as an information source. If the first ChatGPT responses to patient queries happen to be similar in quality and context as those provided by the treating physicians, patients would likely deem the bot to be trustworthy, without realizing that further answers might be of inferior or even very poor quality. Chow et al. (17) pointed out similar concerns regarding ChatGPT’s use as a medical chatbot: as it draws information from the internet, this “disruptive technology” can cause for “questionable and uncontrollable” accuracy and currency of medical information. Contrary to our findings on sarcoma-related responses, Uz and Umay recently evaluated the responses of ChatGPT to frequently searched keywords relating to common rheumatic diseases and found them to be a reliable and useful source of information for patients (19).

A possible reason for this discrepancy is the rarity of sarcomas, compared to the relatively high prevalence of rheumatic disorders. Given that ChatGPT is trained on massive datasets of online available information, incomplete, erroneous, or outdated online data on a specific topic would lead to poorer bot responses. Zade et al. (24) previously analyzed the quality of online resources for orthopedic oncology in 48 websites and found a general lack of quality and accuracy, an issue that has been reported by other studies as well (23). As such, it appears unlikely that ChatGPT will be able to consistently provide high-quality responses to sarcoma-related queries in the foreseeable future.

Our evaluation of different parameters of ChatGPT’s responses demonstrated that the bot achieved its worse scores in the accuracy metric, a finding well in-line with the previously mentioned weaknesses of the artificial intelligence’s sources on a rare disease like sarcoma.

On the other hand, its best scores in our study were documented in the metric “appropriateness for patients.” Our results are in line with the findings of Ayers et al. (14), who performed a blinded study comparing physicians’ and ChatGPT’s responses on public questions asked by patients on a social media forum. The bot’s responses were rated significantly more empathetic than the physicians’ replies and achieved the highest empathy scores on a Likert scale approximately 10 times more often compared to the physicians’ responses (14). The authors concluded that the addition of artificial intelligence assistants to patient messaging workflows appeared to be promising, stressing however the need for human review of generated content for accuracy and potential false or fabricated information (14).

Finally, we were able to show that ChatGPT fared better with general questions and definitions, and considerably worse with treatment-related inquiries. Several other studies have also demonstrated that the quality of the bot’s responses in a specific medical field may vary depending on the complexity of the posed inquiries. Hoch et al. (20) analyzed the accuracy of ChatGPT’s responses to practice multiple choice questions designed for otolaryngology board certification and found significant variations in the rates of correct responses between different subspecialties. The authors suggested that this finding might be explained due to a varying availability and quality of training data in the different categories, with the bot performing better in most common categories and worse in rarer subspecialties with potentially more limited literature data (20). Another study by Jung et al. (21) evaluated the performance of ChatGPT in answering questions from the German state examinations for medical students. While the bot was able to pass both parts of the exam, it fared better with questions on facts and definitions and worse with questions necessitating an understanding of complex relationships and multimodal diagnostics or applied knowledge (21). The importance of a multidisciplinary approach at specialized centers for sarcoma patients has been well documented (3–7), and it is considered a prerequisite for optimal patient care (3). We therefore believe that sarcoma patients should be discouraged from using ChatGPT as a source of information for treatment options and approaches.

In conclusion, the answers ChatGPT provided on a rare disease, such as sarcoma, were found to be of very inconsistent quality, with some answers being classified as very good and others as very poor, depending on the complexity and nature of the question. Taken the extraordinary popularity ChatGPT achieved in only a few months, sarcoma physicians should be aware of the risks of misinformation that ChatGPT poses and advise their patients accordingly. However, given that ChatGPT achieved higher scores in the evaluation of how appropriate its responses are for patients, future studies should evaluate whether it can be used by sarcoma physicians as a supervised tool to better communicate complex aspects of their disease to affected patients.

## Data availability statement

The raw data supporting the conclusions of this article will be made available by the authors, without undue reservation.

## Ethics statement

An approval from our local ethic committee was not required, as the study did not involve human subjects.

## Author contributions

MV: Writing – review & editing, Writing – original draft, Methodology, Investigation, Data curation, Conceptualization. JS: Writing – review & editing, Supervision, Investigation. MS: Writing – review & editing, Formal analysis, Data curation. SS: Writing – review & editing, Supervision. AL: Writing – review & editing, Supervision, Methodology, Investigation, Conceptualization. DA: Writing – review & editing, Validation, Supervision, Methodology, Investigation, Formal analysis, Conceptualization.

## Footnotes

^1^https://help.openai.com/en/articles/7102672-how-can-i-access-gpt-4; https://nerdynav.com/gpt-4-statistics-facts/#how-to-access-gpt4-free-and-paid-methods
